# Bacteriology and Water Examination

**Published:** 1901-05-25

**Authors:** 


### BACTERIOLOGY AND WATER EXAMINATION.

In view of the great prominence so often given to the results obtained by the bacteriological examination of drinking water, and the great faith which many people seem to have in the pronouncements published from time to time as to the number of microbes per cubic centimetre contained in the various specimens of water submitted to such examination, everything which helps us to give bacteriological reports their true value is worthy of the most careful consideration. It is well recognised that of all the microscopic forms of life found in so many samples of water, only the most infinitesimal proportion has any casual relation to disease, and some investigators have gone so far as to say that the free growth of ordinary water organisms forms one of our best protections against the development of pathogenic forms, so that in a multitude of microbes we have safety. It is easy then to see that it is far more important to determine the kind than the number of the organisms present. But the hunt for pathogenic forms has not been very successful. Water which has been known to be dangerous has so often failed to show the presence of disease germs that no bacteriologist would nowadays dare to pronounce as to the innocuous nature of any sample on the ground of its appearing free from recognisable pathogenic forms. Thus the bacteriologist has been reduced to much the same position as the chemist, and although it may happen, now and then, that he may feel justified in asserting that a given sample is infected with a given germ, in the vast majority of instances he has to be content with saying that it shows evidences of previous sewage contamination; these evidences consisting generally of the presence of more than a minimal proportion of certain organisms which are known to be chiefly if not exclusively produced in the intestinal canal. Unfortunately neither the chemist nor the bacteriologist have hitherto been able to say with any great certainty how recent is this "previous sewage contamination." But this is often the very crux of the whole question, for pathogenic organisms are in many cases short-lived, and the danger of the contamination depends largely upon the date at which it took place. Under these circumstances great interest attaches to the researches made by Dr. Houston as to the significance of the presence of streptococci in water, detailed in the recently-issued annual report of the Medical Officer to the Local Government Board; for if, as he shows, these organisms can only maintain their independent existence in water for a short period, their presence may be taken as proof of very recent contamination. The two organisms on the presence of which dependence is mostly placed as proving previous sewage contamination are the B. coli and the B. enteritides sporogenes. The latter, however, being a spore-bearing organism, its presence cannot be taken as evidence of necessarily recent pollution; and, although it may fairly be said that the presence of B. coli in any number in either soil or water implies recent pollution of animal sort, the word "recent" in this regard must be taken as having a somewhat wide meaning. But, says Dr. Houston, is there any microbe or class of microbes which by its presence implies animal pollution of extremely recent, and therefore specially dangerous kind? And he answers that the streptococci are to be thought of in this sense. The position may be put thus: on the one hand in pure water and in the washings of virgin soil he has uniformly failed to detect microbes of the streptococcus class, while on the other hand streptococci are habitually found in such small volumes of crude sewage as one-thousandth part of a cubic centimetre, and are ordinarily abundant in minimal quantities of human excrement. Thus we are led to the belief that the discovery of streptococci in a water sample affords a very valuable indication, not merely that the water has been contaminated by animal organic matter, but that the contamination in question has been of quite recent occurrence and thus of a sort peculiarly dangerous to a water service.

If further experience should show that this test is to be relied upon as an indication of the date of a contamination, and if we are thus enabled to say, not merely that a given sample of water has some time or other been exposed to sewage contamination, but that this contamination has so recently left the human body that if it contained pathogenic organisms these would probably be alive and in full activity, then a great advance will have been made in the position occupied by bacteriology in water examination.

